# Supplementary material for: Integrated evolutionary analysis reveals antimicrobial peptides with limited resistance
Source: Nat Commun. 2019 Oct 4;10:4538. doi: 10.1038/s41467-019-12364-6 (PMC6778101; doi:10.1038/s41467-019-12364-6)
Supplement: Supplementary file 1 — Supplementary Information [file 41467_2019_12364_MOESM1_ESM.pdf]

Supplementary Information

Integrated evolutionary analysis reveals antimicrobial peptides with limited resistance

Spohn and Daruka *et al.*

Supplementary Tables

Supplementary Table 1 –AMPs used in the laboratory evolution experiments and the genomic overexpression screen. The AMPs also used in the metagenomic screen are indicated by asterisk (*).

Antimicrobial peptide		Source	Sequence	3D structure
Abbrev.	Name			
BAC5	Bactenecin 5	Bovine	RFRPPIRRPPIRPPFYPPFRPPIRPPIFPPIRPPFRPPLGPFP	rich in P and R[1]
CAP18	Rabbit 18-kDa cationic antimicrobial protein	Rabbit	GLRKRLRKFRNKIKEKLKKIGQKIQGFVPKLAPRTDY	helix [2]
CP1*	Cecropin P1	Small intestinal roundworms	SWLSKTAKKLENSAKKRISEGIAIAIQGGPR	helix [3]
HBD3	Human beta-defensin -3	Human	GIINTLQKYYRVRGGRAVLSLPKEEQIGKSTRGRKRRKK	combined helix and beta structure [4]
IND*	Indolicidin	Bovine	ILPWKWPWWPWRR	Non helix beta [5]
LL37	LL-37 cathelicidin	Human	LLGDFFRKSKEKIGKEFKRIVQRIKDFLRNLVPRTES	helix [6]
PEX	Pexiganan	Synthetic	GIGKFLKKAKKFGKAFVKILKK	helix [7]
PGLA	Peptide Glycine-Leucine Amide	Frog	GMASKAGAIAGKIAKVALKAL	helix [8]
PLEU	Pleurocidin	Fish	GWGSFFKKAAHVGKHVGKAALTHYL	helix [9]
PR39	PR-39	Pig	RRRPRPPYLPRPRPPPFFPPRLPPRIPPGFPPRFPPRFP	rich in P and R [10]
PROA	Protamine	Fish	TSSPPAAVVVRRRRRRRRRRRRRRRRRRR	rich in R [11]
PXB	Polymyxin B	Bacteria	KTKKKFLKKT	beta turn and/or gamma turn [12]
R8*	R8	Synthetic	FLGKVFKLASKVFKAVFGKV	unknown [13]
TPII*	Tachyplesin II	Crab	RWCFRVCYRGICYRKCR	two disulfide bridges [14]

Supplementary Table 2 – Antibiotics used in the laboratory evolution experiments and the genomic overexpression screen. The antibiotics also used in the metagenomic screen are indicated by asterisk (*).

Antibiotic		Mode of action	Type of action
Name	Abbrev.		
Tetracycline	TET	Protein synthesis, 30S	Bacteriostatic
Doxycycline	DOX	Protein synthesis, 30S	Bacteriostatic
Chloramphenicol	CHL*	Protein synthesis, 50S	Bacteriostatic
Erythromycin	ERY	Protein synthesis, 50S	Bacteriostatic
Trimethoprim	TRM*	Folic acid biosynthesis	Bacteriostatic
Ampicillin	APC	Cell wall	Bactericidal
Cefoxitin	FOX	Cell wall	Bactericidal
Nalidixic acid	NAL	Gyrase	Bactericidal
Ciprofloxacin	CPR*	Gyrase	Bactericidal
Nitrofurantoin	NIT	Multiple mechanisms	Bactericidal
Tobramycin	TOB*	Protein synthesis, 30S, Aminoglycosides	Bactericidal
Kanamycin	KAN	Protein synthesis, 30S, Aminoglycosides	Bactericidal

Supplementary Table 3– Fold change in minimum inhibitory concentration (MIC) as a result of artificial gene amplification. Fold changes were calculated by dividing the MIC provided by the pooled plasmids of the ASKA library (that comprises every *E. coli* ORF cloned into an expression vector) with the MIC of *E. coli* K-12 BW25113 carrying the empty plasmid of the ASKA library. Chloramphenicol (CHL) was excluded from the experiment as it is the selection marker for the ASKA plasmids. Three biological replicates were used. For AMP and antibiotic abbreviations, see Supplementary Table 1 and Supplementary Table 2, respectively.

AMP/AB	MIC value (µg.mL-1) of E. coli K-12 BW25113 carrying the empty plasmid of the ASKA library			MIC value (µg.mL-1) of E. coli K-12 BW25113 carrying the pooled plasmids of the ASKA library			
	Replicate 1	Replicate 2	Replicate 3	Replicate 1	Replicate 2	Replicate 3	Average fold change of the 3 replicates
BAC5	5.79	5.79	5.79	4.82	5.79	4.82	0.89
CAP18	24.11	20.09	24.11	24.11	24.11	24.11	1.07
CP1	2.31	2.31	2.31	2.31	2.31	2.31	1.00
HBD3	195.36	195.36	281.31	405.09	337.58	281.31	1.60
IND	28.93	28.93	28.93	28.93	28.93	28.93	1.00
LL37	14.45	12.04	14.45	14.45	17.34	14.45	1.15
PEX	12.06	17.36	14.47	14.47	17.36	17.36	1.13
PGLA	83.72	83.72	100.47	120.56	120.56	120.56	1.36
PLEU	40.4	88.76	31.08	52.52	40.4	52.52	1.15
PR39	5.79	5.79	5.79	5.79	5.79	5.79	1.00
PROA	126.51	126.51	442.78	126.51	36.15	442.78	0.76
PXB	3.18	3.18	3.18	3.18	3.18	3.18	1.00
R8	11.57	11.57	11.57	11.57	11.57	9.46	0.94
TPII	3.48	3.48	3.48	3.48	3.48	3.48	1.00
APC	4	4	4	256	256	256	64.00
CPR	0.0057	0.0057	0.0057	0.0118	0.0142	0.0142	2.35
DOX	0.56	0.43	0.56	1.59	1.59	1.59	3.14
ERY	10.98	14.28	14.28	18.56	24.13	24.13	1.69
FOX	6.07	6.07	6.07	69.19	69.19	69.19	11.39
KAN	9.65	7.43	7.43	12.55	9.65	12.55	1.43
NAL	0.37	0.37	0.37	1.05	1.05	1.05	2.86
NIT	6.53	6.53	6.53	14.69	14.69	6.53	1.83
TET	0.62	0.62	0.62	1.29	1.07	1.07	1.84
TOB	1.49	1.24	1.49	2.15	2.58	2.58	1.75
TRM	2	2	2	128	64	64	42.67

Supplementary Table 4 – Antimicrobial activity of TPII and CP1 against a set of 20 pathogenic strains.

	Strain		MIC (µM)	
			**CP1**	**TPII**
Gram-negatives	*Escherichia coli*	ATCC 25922	1.2	1.8
Gram-negatives	*Escherichia coli*	ATCC 35218	1.2	1.8
Gram-negatives	*Escherichia coli*	NCTC 13351	0.6-1.2	0.9-1.8
Gram-negatives	*Escherichia coli*	ATCC BAA 2469	1.2	0.9
Gram-negatives	*Escherichia coli*	ATCC BAA 2340	1.2	0.9-1.8
Gram-negatives	*Shigella sonnei*	HNCMB 25021	0.6-1.2	0.9
Gram-negatives	*Shigella flexneri*	HNCMB 20018	1.2	1.8
Gram-negatives	*Enterobacter cloacae* subsp. *cloacae*	ATCC 13047	>9.6	3.5
Gram-negatives	*Salmonella enterica* subsp. *enterica* serovar Typhimurium	LT2	1.2-2.4	1.8
Gram-negatives	*Salmonella enteritidis*	HNCMB 10092	1.2	1.8
Gram-negatives	*Klebsiella pneumoniae*	ATCC 10031	1.2-2.4	0.9-1.8
Gram-negatives	*Klebsiella pneumoniae*	NCTC 13440	1.2	1.8
Gram-negatives	*Klebsiella pneumoniae* subsp. *pneumoniae*	ATCC 700603	2.4	1.8
Gram-negatives	*Acinetobacter baumannii*	ATCC 17978	0.6-1.2	1.8
Gram-negatives	*Pseudomonas aeruginosa*	ATCC 27853	2.4	1.8
Gram-positives	*Enterococcus faecium*	ATCC 700221	>9.6	3.5
Gram-positives	*Streptococcus pyogenes*	ATCC 19653	>9.6	0.9
Gram-positives	*Staphylococcus aureus* subsp. *aureus*	ATCC 25923	>9.6	3.5
Gram-positives	*Staphylococcus aureus* subsp. *aureus*	ATCC 29213	>9.6	3.5
Gram-positives	*Methicillin-resistant Staphylococcus aureus* subsp. *aureus*	ATCC 43300	>9.6	3.5

Supplementary Table 5 –Haemolysis percentage at different AMP concentrations. Optical density (OD) was measured at 565 nm. Melittin (50 µg.mL-1) and TBS buffer were used as positive (100 % haemolysis) and negative (no haemolysis) controls, respectively. Haemolytic effect of each peptide at each concentration was calculated as follows: Haemolysis percentage = (Compound OD_{565nm}-TBS OD_{565nm}) X 100 / (Melittin OD_{565nm} - TBS OD_{565nm}).

	Haemolysis percentage			
Concentration (µg.mL-1)	TPII	CP1	IND	R8
2500	33	4	74	131
1250	21	3	89	139
625	15	2	56	128
312	10	5	14	107
156	6	1	4	55
78	3	1	3	24
39	3	1	2	9
19.5	2	1	2	3
9.75	2	1	2	2
0	1	1	1	2

Supplementary Table 6 -Activity/toxicity index (ATI) of TPII and CP1. ATI is calculated as the ratio between the concentration causing 10% haemolysis (minimum haemolytic concentration, MHC) and the median of MICs (MM) in *E. coli* K12.

	TPII	CP1
MHC (µg.mL-1)	312	>2500
MM (µg.mL-1)	2.01	2.95
ATI	155.22	>847.45

Supplementary Table 7- Effect of incubation time on the minimum inhibitory concentration (MIC) of AMPs. We tested whether the prolonged incubation time can affect the MIC of AMPs. To test this, two sets of **AMP containing** MIC plates were prepared in parallel. The first set was immediately inoculated with *E.coli* K-12 BW25113, while the second set was pre-incubated 72 hours before inoculation with the same strain. We measured the change in the MIC after 24, 48 and 72 hours and no differences were observed in the MIC between freshly prepared and pre-incubated plates.

	MIC (freshly prepared AMP)			MIC (pre-incubated AMP)		
Time allowed for bacterial growth (hours)	24	48	72	24	48	72
TPII (µg.mL-1)	5	5	5	6	6	6
PXB (µg.mL-1)	4.2	4.2	4.2	5	5	5
LL37 (µg.mL-1)	14.5	17.4	17.4	17.4	17.4	17.4
BAC5 (µg.mL-1)	14.5	14.5	14.5	14.5	14.5	14.5

Supplementary Figures

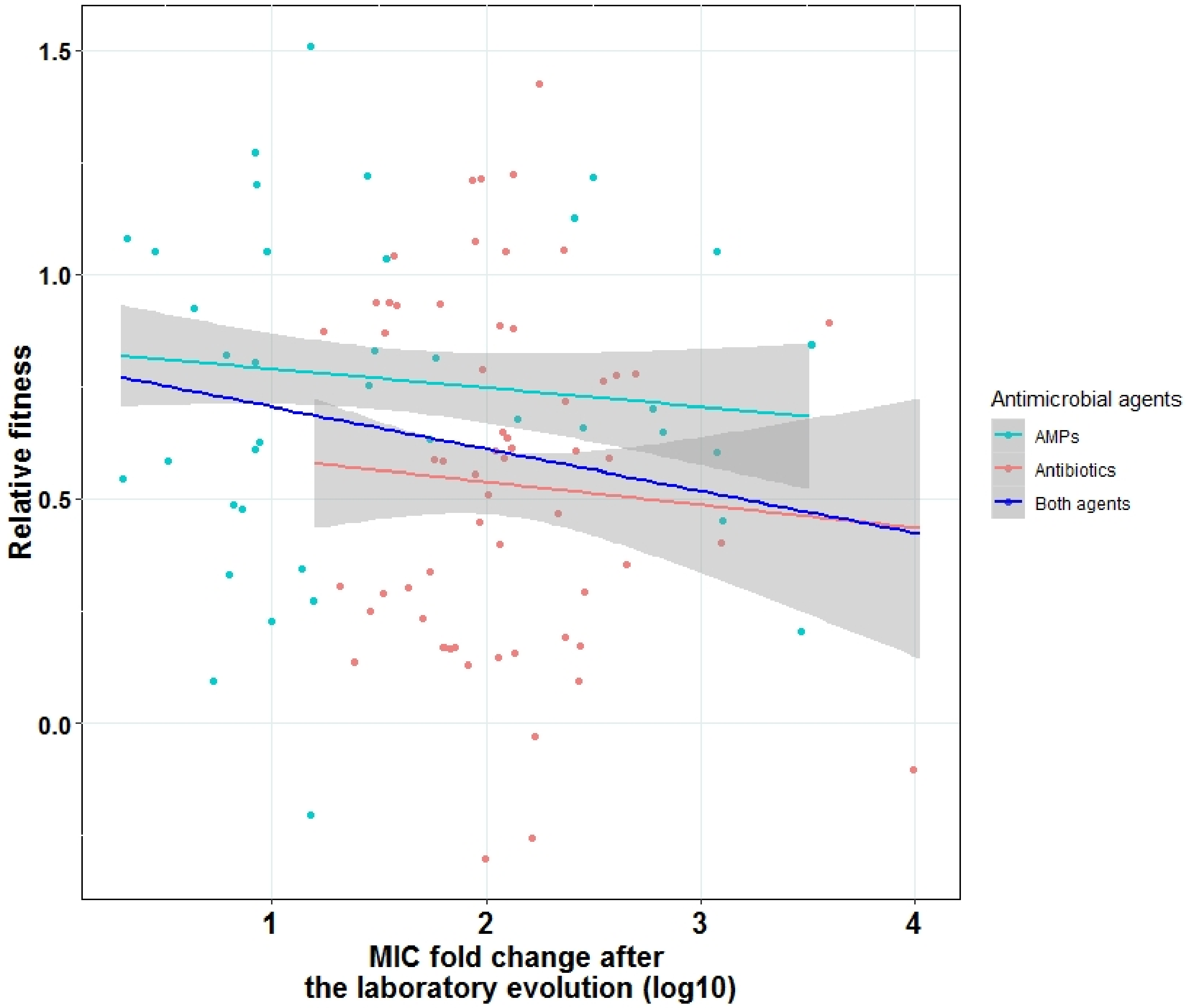

Supplementary Figure 1- Correlation between relative fitness and minimal inhibitory concentration (MIC) fold change. The figure shows a weak correlation between MIC fold change and relative fitness (spearman's rho = -0.24, P = 0.016, (N=98)). The weak correlation disappears when we control for the antimicrobial agents (P = 0.6 and P = 0.39 for antibiotics (N=60) and AMPs (N=38), respectively). Source data are provided as a Source Data file.

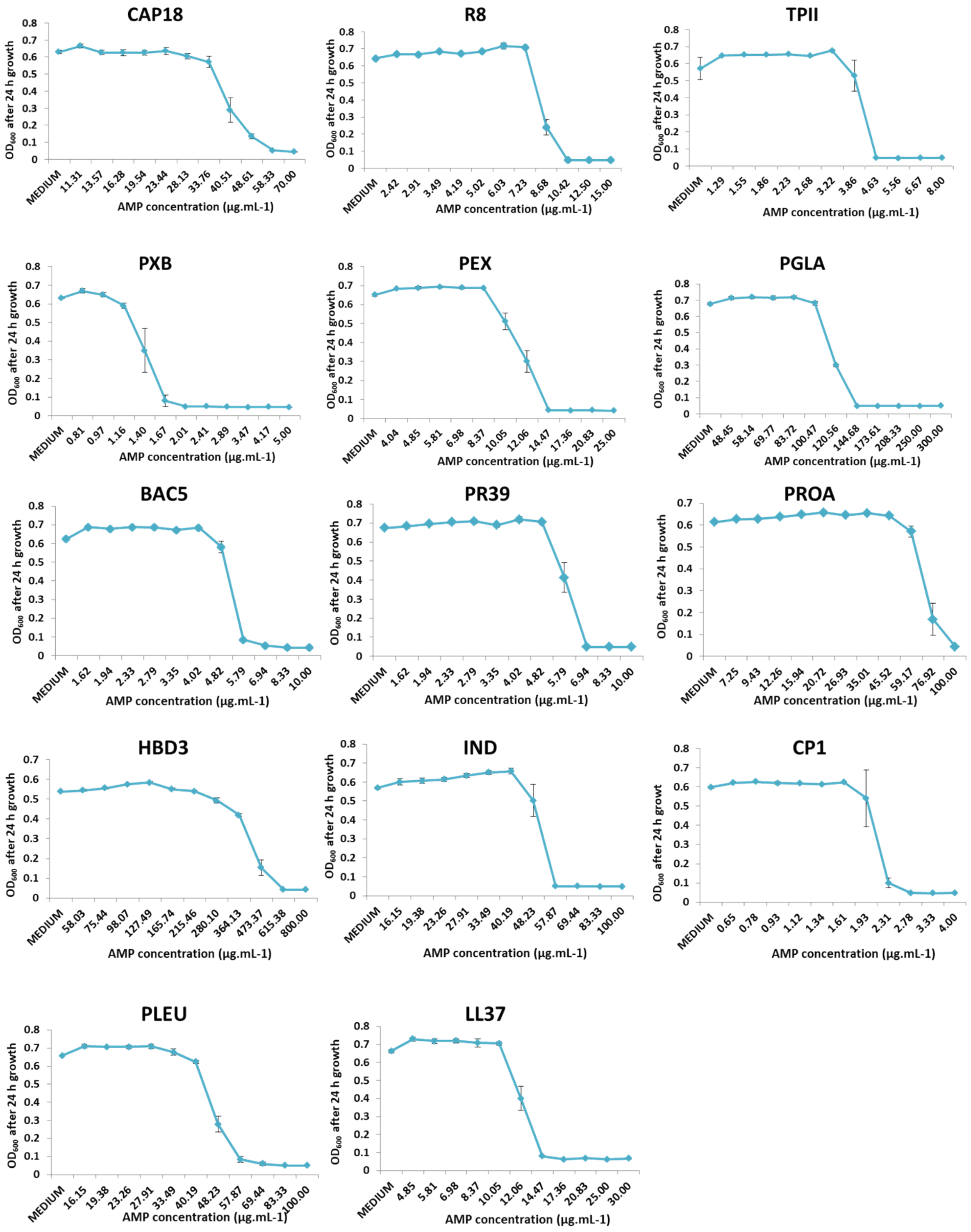

Supplementary Figure 2A- Dose-response curves of *E. coli* K-12 BW25113 strain against AMPs. For abbreviations, see Supplementary Table 1. Each data point shows the mean ± s.e.m. of three biological replicate. Source data are provided as a Source Data file.

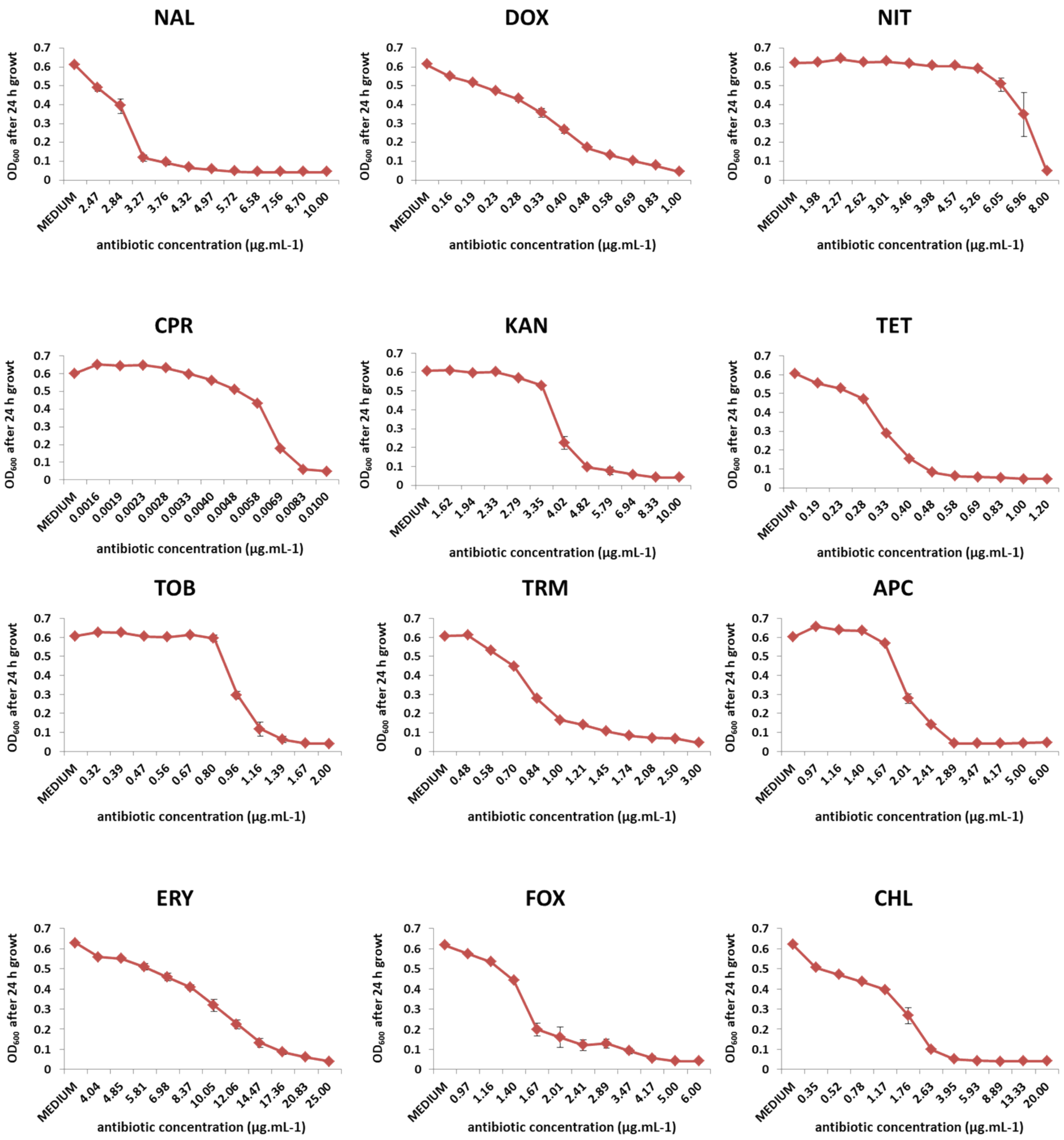

Supplementary Figure 2B- Dose-response curves of *E. coli* K-12 BW25113 strain against antibiotics. For abbreviations see Supplementary Table 2. Each data point shows the mean ± s.e.m. of three biological replicate. Source data are provided as a Source Data file.

Supplementary References

1. Gennaro, R., Skerlavaj, B. & Romeo, D. Purification, composition, and activity of two bactenecins, antibacterial peptides of bovine neutrophils. *Infect. Immun.* **57**, 3142–3146 (1989).

2. Chen, C. *et al.* The solution structure of the active domain of CAP18 - a lipopolysaccharide binding protein from rabbit leukocytes. *FEBS Lett.* **370**, 46–52 (1995).

3. SIPOS, D., ANDERSSON, M. & EHRENBERG, A. The structure of the mammalian antibacterial peptide cecropin P1 in solution, determined by proton-NMR. *Eur. J. Biochem.* **209**, 163–169 (1992).

4. Schibli, D. J. *et al.* The solution structures of the human β-defensins lead to a better understanding of the potent bactericidal activity of HBD3 against Staphylococcus aureus. *J. Biol. Chem.* **277**, 8279–8289 (2002).

5. Rozek, A., Friedrich, C. L. & Hancock, R. E. W. Structure of the bovine antimicrobial peptide indolicidin bound to dodecylphosphocholine and sodium dodecyl sulfate micelles. *Biochemistry* **39**, 15765–15774 (2000).

6. Wang, G. Structures of human host defense cathelicidin LL-37 and its smallest antimicrobial peptide KR-12 in lipid micelles. *J. Biol. Chem.* **283**, 32637–32643 (2008).

7. Gottler, L. M. & Ramamoorthy, A. Structure, membrane orientation, mechanism, and function of pexiganan - A highly potent antimicrobial peptide designed from magainin. *Biochim. Biophys. Acta - Biomembr.* **1788**, 1680–1686 (2009).

8. Bechinger, B., Zasloff, M. & Opella, S. J. Structure and dynamics of the antibiotic peptide PGLa in membranes by solution and solid-state nuclear magnetic resonance spectroscopy. *Biophys. J.* **74**, 981–987 (1998).

9. Syvitski, R. T., Burton, I., Mattatall, N. R., Douglas, S. E. & Jakeman, D. L. Structural characterization of the antimicrobial peptide pleurocidin from winter flounder. *Biochemistry* **44**, 7282–7293 (2005).

10. Agerberth, B. *et al.* Amino acid sequence of PR-39. Isolation from pig intestine of a new member of the family of proline-arginine-rich antibacterial peptides. *Eur. J. Biochem.* **202**, 849–54 (1991).

11. Gatewood, J. M., Schroth, G. P., Schmid, C. W. & Bradbury, E. M. Zinc-induced secondary structure transitions in human sperm protamines. *J. Biol. Chem.* **265**, 20667–72 (1990).

12. Pristovšek, P. & Kidrič, J. Solution structure of polymyxins B and E and effect of binding to lipopolysaccharide: An NMR and molecular modeling study. *J. Med. Chem.* **42**, 4604–4613 (1999).

13. Loose, C., Jensen, K., Rigoutsos, I. & Stephanopoulos, G. A linguistic model for the rational design of antimicrobial peptides. *Nature* **443**, 867–9 (2006).

14. Miyata, T. *et al.* Antimicrobial peptides, isolated from horseshoe crab hemocytes, tachyplesin II, and polyphemusins I and II: chemical structures and biological activity. *J. Biochem.* **106**, 663–8 (1989).
