# Supplementary material for: Integrated evolutionary analysis reveals antimicrobial peptides with limited resistance
Source: Nat Commun. 2019 Oct 4;10:4538. doi: 10.1038/s41467-019-12364-6 (PMC6778101; doi:10.1038/s41467-019-12364-6)
Supplement: Supplementary file 4 — Description of Additional Supplementary Files [file 41467_2019_12364_MOESM4_ESM.pdf]

Description of Additional Supplementary Files

File Name: Supplementary Data 1
Description: Fold change in minimum inhibitory concentration (MIC) of adapted lines following the laboratory evolution.

File Name: Supplementary Data 2
Description: Measured relative fitness of adapted line.

File Name: Supplementary Data 3
Description: Physicochemical characteristics of the 14 AMPs.

File Name: Supplementary Data 4
Description: Cross-resistance interactions of AMP-adapted lines.

File Name: Supplementary Data 5
Description: Mutations identified in the 38 whole-genome sequenced AMP adapted E. coli K-12 BW25113 lines.

File Name: Supplementary Data 6
Description: Mutations identified in AMP -adapted lines that were previously also connected to AMP resistance in the literature.

File Name: Supplementary Data 7
Description: List of resistance contigs identified from the functional selection of the small-insert shotgun soil metagenomic library.

File Name: Supplementary Data 8
Description: Applied AMP/antibiotic concentrations during the laboratory evolution experiments.

File Name: Supplementary Data 9
Description: Oligonucleotides used for reinsertion of single mutations into the wild-type genetic background (E. coli K-12 BW25113).
